# Genetic determinants of micronutrient levels and their causal impact on osteonecrosis of the femoral head: A 2-sample Mendelian randomization study

**DOI:** 10.1097/MD.0000000000042487

**Published:** 2025-05-30

**Authors:** Hai Hu, Rui Huang, Xue Li, Pengfei Liu, Shujun Ren, Yiwei Shen

**Affiliations:** a Department of Orthopaedics,The Second Affiliated Hospital of Heilongjiang University of Chinese Medicine, Harbin, Heilongjiang, China; b Department of Orthopaedics, Li Huili Hospital, Ningbo Medical Center, Ningbo, Zhejiang Province, China; c Department of Orthopaedics, Shanhe YiPa Research Institute, Tianjin, China; d Department of Orthopaedics,The First Affiliated Hospital of Heilongjiang University of Chinese Medicine, Harbin, Heilongjiang, China; e Department of Orthopaedics, Binhai New Area Hospital of TCM. Tianjin (Fourth Teaching Hospital of Tianjin University of TCM), Tianjin, China; f Department of Orthopaedics, Key Laboratory of Northern Medicine Base and Application under Ministry of Education, Harbin, Heilongjiang, China; g Department of Orthopaedics, Key Laboratory of Chinese Materia Medica, Ministry of Education, Heilongjiang University of Chinese Medicine, Harbin, Heilongjiang, China.

**Keywords:** Mendelian randomization, micronutrients, osteonecrosis, potassium, risk reduction

## Abstract

Osteonecrosis of the femoral head (ONFH) is a debilitating condition with unclear pathophysiology. Micronutrients are implicated in bone health, but their causal relationship with ONFH remains uncertain. This study aims to investigate the potential causal relationship between circulating levels of 15 micronutrients and the risk of ONFH using a 2-sample Mendelian randomization (MR) approach. The objective was to determine whether circulating levels of specific micronutrients (copper, calcium, carotene, folate, iron, magnesium, potassium, selenium, vitamin A, vitamin B12, vitamin B6, vitamin C, vitamin D, vitamin E, and zinc) have a causal impact on the risk of ONFH. This study employed a 2-sample MR approach using summary-level data from 15 genome-wide association studies (GWAS) focused on micronutrient exposures and 1 GWAS for ONFH. The study adhered to 3 fundamental MR assumptions and used the inverse variance weighting method as the primary analysis method, supplemented by MR-Egger regression and MR-PRESSO to assess heterogeneity and pleiotropy. To minimize population stratification bias, the study included only individuals of European descent. The primary finding was a significant association between genetically predicted higher levels of potassium and a reduced risk of ONFH (OR = 0.440, 95% confidence interval (CI) 0.2012–0.959, *P* = .039 per 1 standard deviation). No other micronutrients showed significant associations with ONFH risk. Sensitivity analyses, including MR-Egger regression and leave-one-out analysis, confirmed the robustness of these findings. Our findings indicate a significant association between genetically predicted higher potassium levels and a reduced risk of ONFH in individuals of European descent, while no other micronutrients demonstrated significant associations. Sensitivity analyses confirmed these results, suggesting a potential protective role of potassium in ONFH.

## 1. Introduction

Osteonecrosis of the femoral head (ONFH), a condition often linked to disruptions in bone homeostasis, may have its pathogenesis influenced by the intricate interplay of micronutrients, which are essential for maintaining bone health and structure.^[1,2]^ The role of micronutrients in maintaining bone health and preventing osteonecrosis has been extensively studied.^[3–7]^ Micronutrients such as calcium,^[8]^ vitamin C,^[4,9]^ vitamin D,^[10]^ and vitamin E^[4,5]^ are crucial for bone mineralization and remodeling. Calcium serves as the primary mineral component of bone and is essential for maintaining bone strength and density.^[8]^ Vitamin D plays a pivotal role in calcium homeostasis by promoting calcium absorption in the intestines and enhancing calcium deposition in bone. Vitamin D deficiency has been associated with an increased risk of osteonecrosis,^[11]^ and its supplementation could be beneficial in the treatment of osteonecrosis.^[10]^ Additionally, vitamin C has been shown to enhance the structure of osteogenic cell sheets when cultured with bone marrow cells, potentially aiding in bone formation and repair processes affected by osteonecrosis.^[12]^ Furthermore, a combination of vitamin C and magnesium or vitamin E supplementation has been reported to attenuate steroid-associated osteonecrosis in a rat model, highlighting its therapeutic potential.^[4,9]^ Vitamin E, recognized for its antioxidant properties, has demonstrated a protective role in osteonecrosis, particularly in the context of steroid-induced cases.^[4,5,13]^ Vitamin E can prevent the development of osteonecrosis in animal models, reduce osteocyte apoptosis, and mitigate DNA oxidative damage in bone marrow cells.^[4,5,13]^

Beyond these well-established micronutrients, emerging evidence suggests that other micronutrients may also play important roles in bone metabolism and homeostasis. Potassium, for instance, has been shown to influence bone density and structure by modulating the function of osteoblasts and osteoclasts, cells responsible for bone formation and resorption, respectively.^[14,15]^ Zinc plays a crucial role in bone matrix mineralization and osteogenesis, and its deficiency has been linked to impaired bone growth and development.^[16,17]^ Selenium, a vital micronutrient, is recognized for its antioxidant properties, involvement in bone metabolism and cell proliferation, and its protective role against osteonecrosis.^[3,18]^ Biodegradable magnesium implants and magnesium supplementation have been linked to reduced osteonecrosis in animal models, promoting bone healing and angiogenesis.^[9,19]^ Magnesium osteogenic effects may offer a valuable therapeutic approach for treating this condition. In addition, the potential role of copper, carotene, folate, vitamins A, B6, B12, and E in bone health has yet to be thoroughly investigated.

Mendelian randomization (MR) is a powerful analytical tool that utilizes genetic variants as instrumental variables (IVs) to investigate causal relationships between exposures and outcomes.^[20–22]^ MR overcomes the limitations of observational studies by minimizing confounding and reverse causation bias, thereby providing stronger evidence for causal inference.^[20,23,24]^ This study aims to utilize a 2-sample MR approach to investigate the potential causal relationship between circulating levels of 15 micronutrients (copper, calcium, carotene, folate, iron, magnesium, potassium, selenium, vitamin A, vitamin B12, vitamin B6, vitamin C, vitamin D, vitamin E, and zinc) and the risk of ONFH. By leveraging genetic data from large-scale genome-wide association studies (GWAS), this study seeks to identify specific micronutrients that may influence ONFH development and progression, providing valuable insights into the pathophysiology of this condition and potentially identifying novel therapeutic targets.

## 2. Materials and methods

### 2.1. Study design

**Figure 1.**
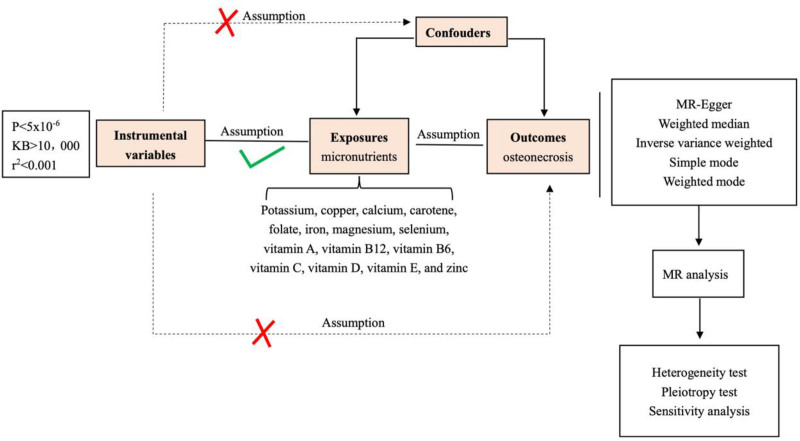
The Mendelian randomization (MR) framework. The MR approach is predicated on 3 foundational assumptions. Initially, single-nucleotide polymorphisms (SNPs) designated as instrumental variables (IVs) must exhibit a robust correlation with the exposure of interest. Subsequently, these chosen SNPs should be devoid of any confounding influences. Ultimately, the instrumental variables should demonstrate a connection to osteonecrosis, the outcome under investigation, solely through the mediation of micronutrients, precluding any direct linkage.

For causal inference through MR studies to be valid, adherence to 3 fundamental assumptions regarding genetic variants is essential. Initially, a significant link must exist between these variants and the exposure variable, namely micronutrients. Subsequently, it is crucial that the genetic variants are uninfluenced by confounders that could alter the exposure-outcome relationship. Finally, the genetic variants should influence the outcome – osteonecrosis – exclusively through the exposure to micronutrients, ruling out any extraneous pleiotropic pathways (as illustrated in Fig. 1).

In this research, we undertook a 2-sample MR analysis, utilizing publicly accessible summary-level data from a total of 15 GWAS, with 15 GWAS dedicated to the exposures and 1 to the outcome. To minimize the bias associated with population stratification, our cohorts for both exposure and outcome were limited to individuals of European descent.^[25]^ The data employed in this study are open to the public, sourced from projects that have secured the necessary participant consent and have undergone ethical review. Consequently, no further ethical review from an institutional board was required for this specific investigation.

### 2.2. Data sources

Data pertaining to dietary exposures were sourced from the IEU Open GWAS initiative, which encompasses a wide range of intake categories: vegetables, meats, beverages, fruits, and staple foods, totaling 18 distinct exposures. Originating from Europe, the dataset spans a substantial participant spectrum, with sample sizes varying significantly from 2603 to an extensive 460,351 individuals. Our selection prioritized datasets characterized by substantial participant numbers and profound genetic sequencing, aligning with the criteria utilized in prior MR analyses.^[26]^ The IEU Open GWAS project procured these GWAS summary statistics either directly or indirectly, leveraging the UK Biobank as a primary resource (https://www.nealelab.is/uk-biobank). Concurrently, summary data on osteonecrosis were derived from the FinnGen biobank study, which is representative of the European genetic lineage. This dataset includes 604 cases and an impressive 209,575 control subjects, and has similarly been employed in previous MR investigations.^[27]^ Table 1 delineates the specifics of both the exposure and outcome datasets incorporated within the scope of this research.

**Table 1:** Summary of GWAS data sources for micronutrient exposures and osteonecrosis outcome in European populations.

Variable	GWAS ID	Race	N	Sex
Exposure				
Copper	ieu-a-1073	European	2603	Males and females
Calcium	ukb-b-8951	European	64,979	Males and females
Carotene	ukb-b-16202	European	64,979	Males and females
Folate	ukb-b-11349	European	64,979	Males and females
Iron	ukb-b-20447	European	64,979	Males and females
Magnesium	ukb-b-7372	European	64,979	Males and females
Potassium	ukb-b-17881	European	64,979	Males and females
Selenium	ieu-a-1077	European	2603	Males and females
Vitamin A	ukb-b-9596	European	460,351	Males and females
Vitamin B12	ukb-b-19524	European	64,979	Males and females
Vitamin B6	ukb-b-7864	European	64,979	Males and females
Vitamin C	ukb-b-19390	European	64,979	Males and females
Vitamin D	ukb-b-18593	European	64,979	Males and females
Vitamin E	ukb-b-6888	European	64,979	Males and females
Zinc	ieu-a-1079	European	2603	Males and females
Outcome				
Osteonecrosis	finn-b-M13_OSTEONECROSIS	European	210,179	Males and females

### 2.3. Selection of osteonecrosis SNPs

MR is grounded in 3 core postulates: correlation, independence, and exclusion restriction.^[28]^ The underlying principle posits that the selected genetic variants are correlated with risk factors but remain uncorrelated with any confounding elements within the risk factor-outcome nexus (independence). Moreover, it is presupposed that these variants do not influence the outcome via pathways that bypass the risk factor in question (exclusion restriction).

In the process of SNP selection, a stringent genome-wide significance threshold of *P* < 5E−06 was applied to pinpoint SNPs with a robust association to micronutrients. To address linkage disequilibrium, SNPs were grouped with parameters set at kb = 10,000 and *r*^2^ = 0.001. Palindromic SNPs were deliberately omitted due to the inherent difficulties in their identification within the GWAS for exposure and outcome, where micronutrients are often aligned in the same direction. The variance in exposure attributable to each SNP was quantified using the *R*^2^ value, while the F statistic was leveraged to gauge the strength of the instrument, thus circumventing the bias associated with weak instruments.^[29,30]^ Ultimately, any missing SNPs in the summary results were substituted with proxy SNPs (*r*^2^ > 0.8), sourced from the LDlink tool, a valuable web-based resource for examining linkage disequilibrium within population groups (LDlink | An Interactive Web Tool for Exploring Linkage Disequilibrium in Population Groups (nih.gov)).^[31]^ By applying these exclusion criteria, we aimed to ensure that our study population and the genetic instruments used were appropriate for the MR analysis, thereby strengthening the validity of our findings.

### 2.4. Statistical analysis

In our primary MR analysis, we applied distinct statistical methods contingent on the number of SNPs linked to each micronutrient. For micronutrients with a single SNP, the Wald ratio was utilized, whereas for those with 2 or more SNPs, the inverse variance weighting (IVW) method was selected as the principal analytical approach.^[24]^ This methodology was employed to explore the potential pathogenic influence of micronutrients on the risk of osteonecrosis. To scrutinize heterogeneity within our primary MR analysis using IVW, a Cochrane Q test was conducted. While the majority of the outcomes indicated no significant heterogeneity (*P* > .05), some did present heterogeneity. It is crucial to acknowledge that IVW, being our primary method, might inherently encompass heterogeneity, and thus, heterogeneity in specific outcomes does not substantially affect the overall inference of causality.^[24]^

Further to assess causality and to probe for potential pleiotropy, we conducted supplementary evaluations, including MR-Egger regression and MR-PRESSO.^[32]^ Additionally, a leave-one-out analysis was implemented to assess the impact of each individual SNP on the overall MR analysis. To mitigate possible confounding influences, PhenoScanner was utilized to explore any dimorphic phenotypes associated with the individual SNPs under investigation.

Significant causal evidence was established when the following criteria were satisfied: the IVW MR results withstood multiple comparisons with a *q*-value of <0.2 after false discovery rate adjustment; the outcomes from other MR methods were consistent in magnitude and direction with IVW; there was an absence of heterogeneity and directional pleiotropy post-exclusion of potential outliers; and the *P*-value was 2-sided. The MR and associated analyses were executed using the “TwoSampleMR” R package in the R environment (version 4.3.1). Concurrently, when the IVW MR *P*-value was <.05, the other MR methods mirrored the direction of IVW, and there was no heterogeneity or directional pleiotropy, the findings were deemed to potentially suggest causality.

## 3. Result

Table 1 delineates the extensive particulars of the research and the dataset employed. It is noteworthy that the study exclusively included individuals of European ancestry, which was a purposeful selection to reduce the variance in results that could stem from ethnic disparities. This strategic inclusion fosters a uniform group of participants, thereby augmenting the study’s validity by averting the interference of confounding elements linked to ethnic heterogeneity.

### 3.1. Selection of IVs

Genetic variants were screened against screening criteria (*P* < 5E−06, *r*^2^ < 0.001, kb = 10,000) as described previously. Subsequently, we meticulously excluded SNPs influenced by confounding factors and eliminated outliers through the application of the MR-PRESSO technique. The *F*-statistics for the IVs in our final analysis all exceeded 10, signifying a minimal probability of bias due to weak instruments. The impact of micronutrients on osteonecrosis is encapsulated in Table 2 and Table S1, Supplemental Digital Content, https://links.lww.com/MD/P32, which detail the chromosomal position, associated gene, effect allele, alternative allele, and the frequency of the effect allele (EAF). Furthermore, these tables provide an estimation of the relationship between each SNP and inflammatory cytokines as well as osteonecrosis, encompassing beta coefficients, standard errors, and *P*-values.

**Table 2:** Genetic associations of SNPs with serum micronutrient levels and osteonecrosis risk in 2-sample MR analysis: chromosomal positions, effect alleles, and statistical significance.

Exposure	SNP	chr	Effect allele	Other allele	EAF	SNP-micronutrients association			SNP-osteonecrosis association		
						Beta	SE	pval	beta	SE	pval
Copper	rs10014072	4	G	A	0.672	−0.164	0.034	1.13E−06	0.0227302	0.038	.552
Copper	rs1175550	1	G	A	0.219	0.198	0.032	5.03E−10	0.0857442	0.04	.034
Copper	rs12153606	5	T	G	0.194	−0.159	0.034	2.50E−06	0.0133223	0.049	.787
Copper	rs12582659	12	C	T	0.027	1.262	0.27	2.86E−06	−0.0827239	0.076	.276
Copper	rs2769264	1	G	T	0.161	0.313	0.034	2.63E−20	−0.027417	0.048	.571
Copper	rs3857536	6	T	C	0.552	−0.129	0.028	4.08E−06	0.0695299	0.036	.055
Selenium	rs10023369	4	A	G	0.497	−0.144	0.029	4.42E−07	−0.0247014	0.036	.495
Selenium	rs11779526	8	T	A	0.294	0.153	0.032	1.68E−06	−0.0230842	0.038	.544
Selenium	rs3785832	17	C	T	0.404	−0.146	0.031	1.82E−06	−0.0239189	0.036	.511
Selenium	rs4950779	1	C	T	0.034	0.673	0.132	3.16E−07	−0.0100502	0.094	.915
Selenium	rs7163368	15	C	T	0.237	0.158	0.034	4.00E−06	−0.0333771	0.044	.445
Selenium	rs7700970	5	T	C	0.317	0.265	0.037	7.17E−13	−0.0142897	0.042	.733
Vitamin B6	rs10138490	14	C	T	0.061	−0.053	0.011	2.80E−06	0.212903	0.07	.003
Vitamin B6	rs12198456	6	T	C	0.019	0.092	0.02	3.10E−06	−0.0617829	0.202	.76
Vitamin B6	rs12226112	11	T	G	0.341	0.028	0.006	7.50E−07	0.0151405	0.037	.68
Vitamin B6	rs12412051	10	C	G	0.034	0.071	0.015	2.20E−06	−0.0487269	0.088	.581
Vitamin B6	rs141933624	4	A	G	0.021	−0.09	0.019	3.30E−06	0.380225	0.134	.005
Vitamin B6	rs155599	2	C	T	0.705	0.034	0.006	1.00E−08	−0.0249231	0.04	.536
Vitamin B6	rs183178622	4	T	C	0.018	−0.099	0.021	1.70E−06	−0.170008	0.14	.224
Vitamin B6	rs188211816	1	A	G	0.029	−0.079	0.016	1.40E−06	0.157097	0.093	.091
Vitamin B6	rs34938615	14	G	A	0.011	−0.122	0.026	3.90E−06	−0.108726	0.32	.734
Vitamin B6	rs361294	11	C	A	0.691	−0.027	0.006	4.30E−06	−0.0716152	0.04	.074
Vitamin B6	rs3745438	19	C	T	0.034	−0.071	0.016	4.90E−06	−0.0364251	0.059	.534
Vitamin B6	rs3772928	3	C	T	0.575	−0.029	0.006	1.30E−07	−0.002646	0.037	.944
Vitamin B6	rs67450584	22	T	C	0.158	0.037	0.007	8.60E−07	−0.102382	0.056	.066
Vitamin B6	rs7205927	16	C	A	0.412	−0.026	0.006	3.20E−06	0.0375657	0.037	.307
Vitamin B6	rs74640671	8	T	C	0.011	−0.127	0.027	3.40E−06	−0.073665	0.259	.776
Vitamin B6	rs77806858	5	C	T	0.07	−0.051	0.011	1.90E−06	0.00164527	0.079	.983
Vitamin B6	rs9560457	13	T	C	0.404	0.026	0.006	4.10E−06	0.00518803	0.037	.889
Vitamin A	rs10248388	7	C	A	0.048	0.003	0.001	3.40E−06	0.0481809	0.07	.49
Vitamin A	rs1478684	11	G	T	0.229	0.002	0	1.40E−06	−0.0208283	0.045	.646
Vitamin A	rs1953042	9	C	T	0.164	0.002	0	2.60E−06	0.0380547	0.047	.417
Vitamin A	rs2581667	18	G	A	0.858	0.002	0	2.70E−06	−0.0396269	0.058	.492
Vitamin A	rs4131899	3	T	C	0.517	−0.001	0	1.70E−06	−0.084422	0.036	.02
Vitamin A	rs4379489	8	G	A	0.213	0.002	0	4.30E−06	0.0102403	0.041	.804
Vitamin A	rs6005584	22	C	G	0.089	−0.002	0.001	4.40E−06	0.102365	0.058	.077
Vitamin A	rs72774943	2	T	C	0.061	−0.003	0.001	4.80E−06	0.120752	0.082	.139
Vitamin A	rs72833036	6	G	A	0.062	0.003	0.001	3.10E−08	−0.0785789	0.083	.347
Vitamin A	rs7330997	13	C	T	0.134	−0.002	0	1.60E−06	0.0115388	0.066	.861
Vitamin A	rs9982066	21	A	C	0.489	0.001	0	5.00E−07	0.00592583	0.036	.87
Vitamin D	rs10469075	18	T	C	0.189	−0.032	0.007	3.70E−06	0.0274219	0.049	.578
Vitamin D	rs117693112	7	A	G	0.041	0.068	0.014	2.40E−06	−0.102409	0.138	.457
Vitamin D	rs17301981	9	C	T	0.088	−0.044	0.01	4.90E−06	−0.13718	0.064	.033
Vitamin D	rs2399949	3	C	T	0.185	−0.034	0.007	1.20E−06	0.016873	0.049	.732
Vitamin D	rs35775421	14	A	G	0.053	−0.056	0.012	4.60E−06	0.00194495	0.062	.975
Vitamin D	rs4395237	2	G	T	0.05	−0.06	0.013	2.50E−06	0.0557187	0.071	.43
Vitamin D	rs57038272	5	T	C	0.177	0.033	0.007	4.10E−06	−0.0881742	0.044	.047
Vitamin D	rs582962	6	A	G	0.684	−0.028	0.006	3.20E−06	0.0172247	0.037	.644
Vitamin D	rs61942184	12	C	G	0.028	0.082	0.018	3.70E−06	0.0889816	0.132	.5
Vitamin D	rs679830	7	C	T	0.949	−0.06	0.013	2.40E−06	0.00956989	0.079	.903
Vitamin D	rs74593039	10	C	G	0.158	0.035	0.008	3.40E−06	−0.016467	0.051	.747
Vitamin D	rs75713989	3	T	C	0.13	0.039	0.008	1.60E−06	0.0308202	0.057	.588
Vitamin D	rs80261862	7	T	C	0.098	−0.045	0.009	1.30E−06	−0.0725615	0.047	.122
Zinc	rs10484100	14	G	A	0.09	−0.209	0.045	3.30E−06	−0.0021484	0.07	.976
Zinc	rs10931753	2	C	G	0.664	−0.129	0.028	4.94E−06	−0.0383788	0.04	.332
Zinc	rs11232535	11	C	T	0.055	0.325	0.065	6.73E−07	−0.0923327	0.093	.319
Zinc	rs11763353	7	G	A	0.166	−0.192	0.039	6.90E−07	0.00406643	0.043	.925
Zinc	rs1532423	8	G	A	0.629	−0.178	0.026	6.40E−12	0.0175821	0.038	.647
Zinc	rs2120019	15	C	T	0.208	−0.287	0.033	1.55E−18	0.0416717	0.044	.347
Zinc	rs4333127	4	A	G	0.903	0.218	0.047	3.00E−06	0.0675103	0.071	.34
Zinc	rs7148590	14	A	G	0.484	−0.14	0.026	1.37E−07	−0.0235185	0.037	.522
Potassium	rs10764330	10	G	A	0.699	0.028	0.006	2.60E−06	−0.0353363	0.039	.371
Potassium	rs114300683	5	A	G	0.012	−0.115	0.025	4.00E−06	0.305708	0.311	.326
Potassium	rs12296227	12	T	G	0.267	0.028	0.006	3.80E−06	−0.045226	0.049	.353
Potassium	rs12412051	10	C	G	0.034	0.073	0.015	1.20E−06	−0.0487269	0.088	.581
Potassium	rs145857065	5	C	T	0.03	−0.083	0.017	1.60E−06	0.289632	0.158	.067
Potassium	rs148244439	5	T	C	0.052	0.057	0.012	3.90E−06	−0.0740218	0.096	.439
Potassium	rs2745938	1	T	G	0.647	0.026	0.006	4.10E−06	−0.0089334	0.04	.825
Potassium	rs35579431	8	T	C	0.266	0.029	0.006	2.00E−06	−0.0447122	0.038	.241
Potassium	rs361294	11	C	A	0.691	−0.028	0.006	2.00E−06	−0.0716152	0.04	.074
Potassium	rs3772928	3	C	T	0.575	−0.027	0.006	1.50E−06	−0.002646	0.037	.944
Potassium	rs7040926	9	C	T	0.065	−0.051	0.011	4.40E−06	0.069171	0.055	.204
Potassium	rs7479680	11	C	A	0.144	−0.038	0.008	9.00E−07	0.0270097	0.052	.603
Potassium	rs77126457	8	A	G	0.014	0.105	0.023	4.80E−06	−0.0718506	0.218	.742
Potassium	rs77824658	14	G	A	0.09	0.044	0.01	4.40E−06	−0.0356032	0.07	.609
Vitamin B12	rs10924919	1	T	C	0.394	−0.029	0.006	3.90E−07	0.0400637	0.037	.285
Vitamin B12	rs112961770	1	C	G	0.023	−0.089	0.018	1.40E−06	0.274449	0.072	0
Vitamin B12	rs12776611	10	A	G	0.022	−0.088	0.019	3.40E−06	0.131455	0.124	.291
Vitamin B12	rs1419875	10	G	T	0.201	−0.032	0.007	3.90E−06	−0.0036128	0.042	.932
Vitamin B12	rs148901823	7	G	A	0.081	−0.049	0.01	1.30E−06	−0.110139	0.064	.086
Vitamin B12	rs388561	22	C	T	0.881	0.04	0.009	2.50E−06	0.033354	0.053	.531
Vitamin B12	rs61994378	14	C	T	0.028	0.093	0.02	2.30E−06	0.00069522	0.112	.995
Vitamin B12	rs67568068	5	C	T	0.213	−0.032	0.007	1.80E−06	0.0700675	0.04	.079
Calcium	rs11030416	11	A	G	0.042	−0.065	0.014	1.50E−06	0.0331526	0.113	.768
Calcium	rs11088797	21	A	G	0.114	0.04	0.008	3.10E−06	0.00643244	0.048	.893
Calcium	rs117456360	9	A	G	0.043	0.063	0.014	4.40E−06	0.00551735	0.178	.975
Calcium	rs1219820	10	T	C	0.029	−0.076	0.016	1.70E−06	−0.105244	0.109	.336
Calcium	rs12618785	2	G	C	0.399	0.027	0.006	1.20E−06	0.00294815	0.038	.938
Calcium	rs1714800	8	C	G	0.662	0.028	0.006	1.00E−06	−0.107105	0.038	.005
Calcium	rs17712285	18	G	A	0.034	−0.071	0.015	1.70E−06	−0.364816	0.144	.011
Calcium	rs1974821	19	A	G	0.152	0.036	0.008	2.30E−06	0.00427688	0.051	.933
Calcium	rs2443773	8	G	T	0.506	0.027	0.005	5.10E−07	0.019023	0.036	.6
Calcium	rs35683760	16	G	A	0.229	−0.031	0.006	1.50E−06	−0.0412778	0.041	.315
Calcium	rs39308	7	A	G	0.265	0.03	0.006	1.50E−06	0.0823117	0.039	.034
Calcium	rs4535437	5	G	A	0.245	0.032	0.006	4.50E−07	0.0314436	0.04	.429
Calcium	rs4988235	2	A	G	0.723	0.032	0.006	7.40E−08	−0.0355597	0.037	.342
Calcium	rs62347998	5	T	C	0.042	0.064	0.014	3.40E−06	−0.0480846	0.103	.64
Calcium	rs73238581	4	A	C	0.347	−0.027	0.006	2.00E−06	0.00892081	0.038	.816
Calcium	rs7464794	8	C	T	0.499	−0.026	0.005	9.20E−07	0.00470289	0.036	.897
Calcium	rs753899	15	C	T	0.092	0.046	0.009	7.40E−07	−0.0115971	0.06	.847
Calcium	rs8067154	17	A	G	0.219	−0.03	0.007	4.60E−06	−0.0052864	0.045	.906
Calcium	rs8109178	19	T	A	0.121	0.039	0.008	3.00E−06	−0.0368607	0.056	.507
Vitamin E	rs111306778	13	A	G	0.09	−0.048	0.01	5.40E−07	−0.102602	0.059	.084
Vitamin E	rs12165526	22	A	T	0.101	0.048	0.009	1.80E−07	−0.0293039	0.061	.628
Vitamin E	rs12421920	11	G	A	0.094	−0.043	0.009	3.70E−06	0.0622522	0.083	.455
Vitamin E	rs12899673	15	A	G	0.333	0.027	0.006	3.80E−06	0.0821189	0.038	.029
Vitamin E	rs2723979	7	G	T	0.584	−0.027	0.006	1.50E−06	−0.0020506	0.037	.955
Vitamin E	rs35218694	17	G	A	0.034	−0.074	0.015	1.30E−06	−0.177942	0.128	.163
Vitamin E	rs4903544	14	T	C	0.3	−0.03	0.006	9.50E−07	0.0326938	0.039	.397
Vitamin E	rs536912	1	A	C	0.736	0.03	0.006	9.00E−07	−0.0376865	0.041	.364
Vitamin E	rs6033	1	G	A	0.072	−0.052	0.011	9.90E−07	0.00450696	0.058	.938
Vitamin E	rs71385328	17	G	A	0.011	0.13	0.026	7.00E−07	0.0582485	0.078	.453
Vitamin E	rs79966958	9	T	C	0.013	−0.117	0.025	2.00E−06	0.0537545	0.136	.693
Vitamin E	rs979218	7	C	A	0.098	−0.043	0.009	3.10E−06	0.00810737	0.049	.869
Magnesium	rs111419911	20	G	A	0.31	−0.029	0.006	6.30E−07	0.0389965	0.041	.341
Magnesium	rs114575778	4	A	G	0.01	−0.145	0.027	1.20E−07	0.0619252	0.184	.737
Magnesium	rs114989460	6	C	T	0.018	0.096	0.021	4.60E−06	−0.101966	0.131	.435
Magnesium	rs116028267	5	G	C	0.038	−0.067	0.014	3.90E−06	0.0438001	0.083	.599
Magnesium	rs116740989	2	A	C	0.012	0.118	0.025	2.80E−06	−0.0699637	0.12	.558
Magnesium	rs116979507	11	T	C	0.039	0.064	0.014	4.60E−06	0.0328449	0.124	.792
Magnesium	rs1247081	10	T	G	0.508	0.026	0.005	1.70E−06	0.0163021	0.036	.655
Magnesium	rs144862520	7	T	C	0.041	−0.07	0.015	4.70E−06	−0.0339509	0.104	.744
Magnesium	rs147150587	12	A	G	0.01	0.128	0.028	3.40E−06	−0.320578	0.254	.207
Magnesium	rs1559583	18	C	T	0.785	−0.03	0.007	3.50E−06	0.0283239	0.044	.517
Magnesium	rs2745938	1	T	G	0.647	0.027	0.006	1.60E−06	−0.0089334	0.04	.825
Magnesium	rs4535437	5	G	A	0.245	0.03	0.006	1.20E−06	0.0314436	0.04	.429
Magnesium	rs573905	11	G	A	0.547	0.028	0.005	3.20E−07	0.0162808	0.036	.655
Magnesium	rs7022555	9	T	C	0.012	−0.121	0.025	1.80E−06	−0.38724	0.251	.122
Magnesium	rs7339029	13	T	G	0.134	0.036	0.008	4.70E−06	−0.0876174	0.058	.133
Magnesium	rs76330086	9	T	C	0.015	−0.102	0.022	4.40E−06	−0.124031	0.151	.41
Magnesium	rs77126457	8	A	G	0.014	0.115	0.023	4.50E−07	−0.0718506	0.218	.742
Vitamin C	rs114598078	3	T	C	0.042	0.066	0.014	1.90E−06	−0.0281797	0.098	.774
Vitamin C	rs11650824	17	A	T	0.035	0.079	0.016	5.60E−07	−0.0610024	0.069	.378
Vitamin C	rs17482258	12	T	C	0.099	0.043	0.009	3.70E−06	0.0256075	0.057	.655
Vitamin C	rs1883993	22	A	G	0.095	0.045	0.009	1.50E−06	0.0283618	0.044	.522
Vitamin C	rs2018201	12	G	T	0.027	−0.081	0.017	2.50E−06	0.108106	0.147	.463
Vitamin C	rs4238567	15	C	T	0.522	0.025	0.006	4.30E−06	−0.0485348	0.037	.184
Vitamin C	rs4481190	3	C	A	0.351	−0.031	0.006	9.60E−08	0.0320447	0.038	.395
Vitamin C	rs61868302	10	T	C	0.061	−0.057	0.012	1.40E−06	0.0333951	0.095	.726
Vitamin C	rs7626478	3	A	G	0.72	0.028	0.006	4.50E−06	−0.0019432	0.042	.963
Vitamin C	rs9540734	13	A	G	0.478	−0.026	0.005	2.30E−06	−0.0277463	0.036	.445
Iron	rs114738685	11	T	C	0.056	0.055	0.012	2.40E−06	0.0455939	0.073	.531
Iron	rs116863411	20	T	A	0.018	−0.095	0.02	3.50E−06	−0.0217781	0.116	.851
Iron	rs118189684	11	C	T	0.055	0.059	0.012	8.90E−07	−0.0346564	0.105	.742
Iron	rs1370102	10	A	C	0.431	−0.025	0.005	3.80E−06	0.00636435	0.036	.861
Iron	rs148244439	5	T	C	0.052	0.059	0.012	1.30E−06	−0.0740218	0.096	.439
Iron	rs155599	2	C	T	0.705	0.03	0.006	3.40E−07	−0.0249231	0.04	.536
Iron	rs17257441	1	T	C	0.126	−0.038	0.008	4.70E−06	0.01924	0.048	.689
Iron	rs2647238	4	C	T	0.587	−0.025	0.005	3.60E−06	0.0301481	0.037	.412
Iron	rs56256289	7	T	C	0.187	0.033	0.007	2.30E−06	−0.027603	0.041	.503
Iron	rs6463742	7	C	T	0.484	0.026	0.006	2.60E−06	0.0249259	0.037	.498
Iron	rs9297943	8	G	T	0.642	0.027	0.006	2.40E−06	−0.0227664	0.038	.55
Carotene	rs117731008	22	A	G	0.017	0.098	0.021	4.20E−06	−0.0288097	0.097	.766
Carotene	rs12126792	1	G	A	0.012	−0.135	0.028	1.50E−06	−0.210844	0.204	.302
Carotene	rs13295574	9	A	G	0.303	−0.028	0.006	3.70E−06	0.0651263	0.038	.083
Carotene	rs16898247	8	A	G	0.019	−0.107	0.02	9.00E−08	−0.0496164	0.177	.779
Carotene	rs17800766	10	C	T	0.012	−0.122	0.025	1.80E−06	−0.27078	0.26	.298
Carotene	rs1936052	1	T	C	0.156	−0.036	0.008	2.50E−06	0.00835875	0.054	.876
Carotene	rs2998143	10	G	A	0.604	−0.028	0.006	1.80E−06	−0.0067856	0.039	.861
Carotene	rs366337	19	G	A	0.937	0.054	0.011	1.30E−06	−0.0087253	0.091	.924
Carotene	rs3829931	10	A	T	0.973	0.083	0.018	3.00E−06	−0.119552	0.084	.156
Carotene	rs4771831	13	A	G	0.352	−0.027	0.006	4.20E−06	−0.0444677	0.041	.283
Carotene	rs5760695	22	C	T	0.087	0.047	0.01	3.20E−06	0.0149976	0.047	.751
Carotene	rs62417408	6	G	A	0.038	−0.069	0.015	2.40E−06	−0.0167773	0.076	.825
Carotene	rs6596473	5	C	G	0.299	0.028	0.006	3.70E−06	−0.01086	0.039	.781
Carotene	rs6660246	1	C	A	0.45	−0.027	0.006	1.40E−06	−0.0092882	0.037	.799
Carotene	rs77547747	4	C	T	0.056	−0.056	0.012	2.40E−06	−0.0338149	0.107	.752
Folate	rs148031795	18	T	C	0.015	0.104	0.022	3.10E−06	−0.0703335	0.106	.509
Folate	rs1502443	16	G	C	0.63	0.026	0.006	4.30E−06	−0.0700772	0.038	.068
Folate	rs16956822	17	A	G	0.026	−0.079	0.017	3.90E−06	0.0293424	0.079	.709
Folate	rs2449166	8	T	C	0.471	0.025	0.005	4.00E−06	−0.041825	0.037	.259
Folate	rs3772928	3	C	T	0.575	−0.027	0.006	8.20E−07	−0.002646	0.037	.944
Folate	rs7074988	10	G	A	0.064	−0.051	0.011	4.00E−06	0.0498576	0.084	.554
Folate	rs76630415	7	G	T	0.212	−0.037	0.007	2.40E−08	0.0324838	0.045	.468
Folate	rs76802001	22	A	G	0.036	−0.068	0.015	4.60E−06	−0.142896	0.119	.232
Folate	rs78074774	2	T	C	0.045	0.06	0.013	4.90E−06	0.0689563	0.083	.408
Folate	rs79748722	22	T	C	0.028	−0.076	0.016	4.40E−06	−0.003868	0.163	.981
Folate	rs79975477	20	T	C	0.031	0.073	0.016	2.80E−06	0.0373216	0.069	.586
Folate	rs8085166	18	G	A	0.677	0.028	0.006	1.70E−06	−0.0086463	0.038	.818

chr = chromosome, EAF = effect allele frequency, pval = *P*-value, SE = standard error, SNPs = single-nucleotide polymorphisms.

### 3.2. Causal relationship with osteonecrosis

**Figure 2.**
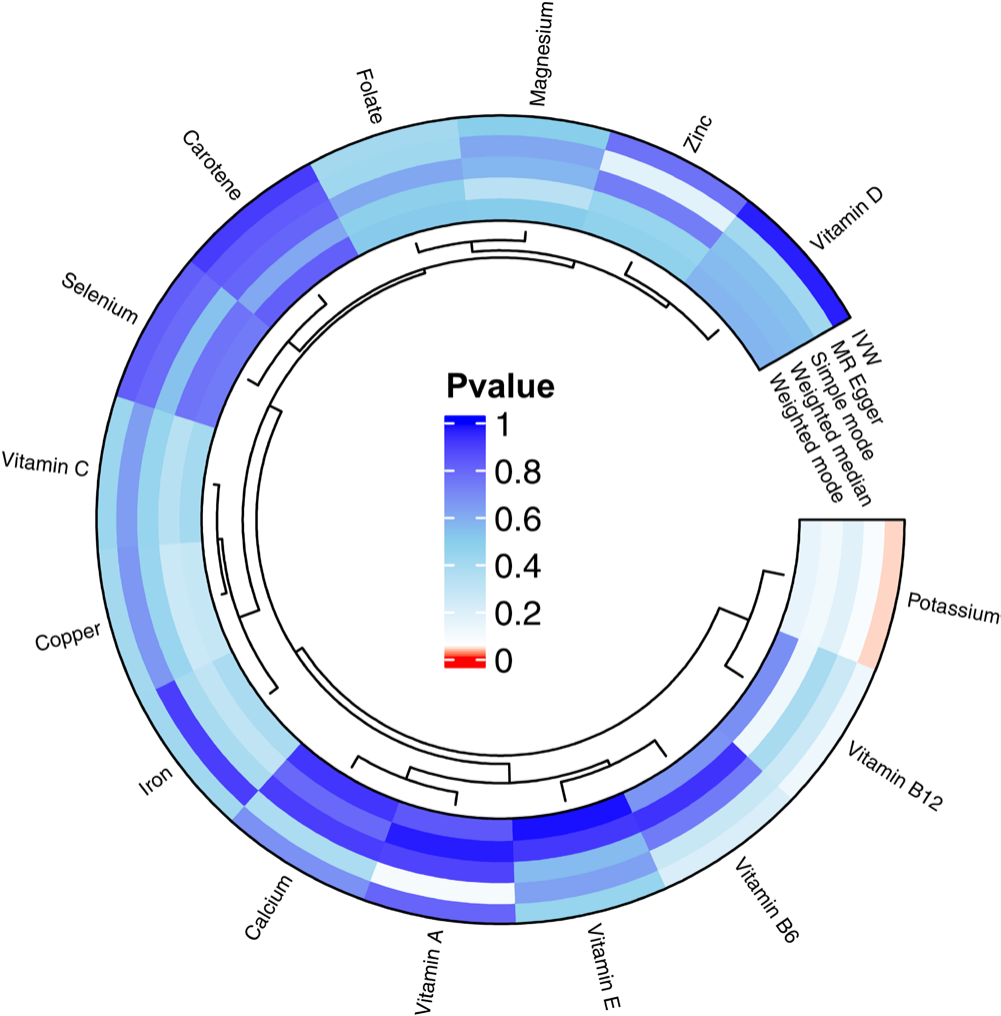
Causal correlations of 15 micronutrients on osteonecrosis. IVW = inverse variance weighted.

**Figure 3.**
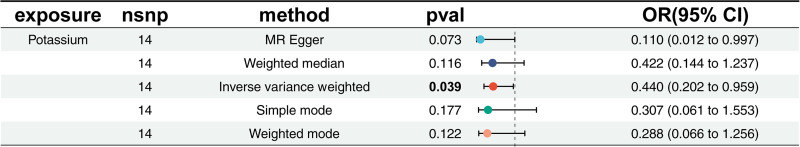
Causal estimation of potassium on osteonecrosis. Forest plots depict the causal estimates of potassium on osteonecrosis. The odds ratio was estimated using the random effects IVW method. Horizontal bars indicate 95% confidence intervals.

Our research indicates a possible role of potassium in the development of osteonecrosis, as evidenced by the IVW method (refer to Fig. 2). Utilizing the IVW method, we observed that elevated genetic prediction levels of potassium correlated with a decreased risk of osteonecrosis (OR = 0.440, 95% CI = 0.2012–0.959, *P* = .039 per 1 standard deviation, refer to Fig. 3). However, we did not detect any associations between osteonecrosis risk and copper, calcium, carotene, folate, iron, magnesium, selenium, vitamin A, vitamin B12, vitamin B6, vitamin C, vitamin D, vitamin E, and zinc (refer to Fig. 2, Fig. S1, Supplemental Digital Content, https://links.lww.com/MD/P33, and Tables S2–S4, Supplemental Digital Content, https://links.lww.com/MD/P32).

### 3.3. Sensitivity analysis

**Figure 4.**
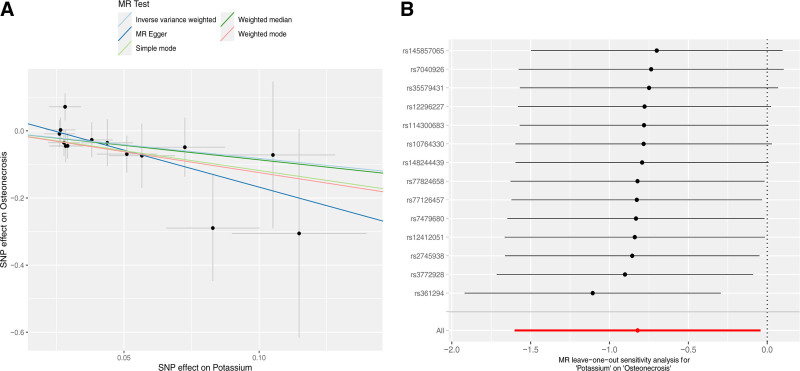
(A) Scatter plot of MR analysis. Scatter plot of genetic correlation between potassium and osteonecrosis by different MR analysis methods. (B) Leave-one-out sensitivity analysis between potassium and osteonecrosis. The black dots and bars indicate the causal estimate and 95% CI using each SNP. The red dot and bar indicate the overall estimate and 95% CI meta-analyzed by the random effects IVW method. IVW = Inverse Variance Weighted.

To evaluate the consistency of effects across studies, we conducted MR-Egger regression and IVW analyses, which revealed the presence of heterogeneity among the studies as detailed in Tables S2 to S4, Supplemental Digital Content, https://links.lww.com/MD/P32. Consequently, we opted for the random effects IVW method as our primary mode of analysis. The intercept from the MR-Egger regression analysis did not suggest the presence of horizontal pleiotropy, as illustrated in Fig. 4A. Cochran Q test did not reveal any heterogeneity (*P* = .736), and directional polymorphisms were absent (MR-Egger-intercept = 0.053, *P* = .213 for MR-Egger-intercept; *P* = .777 for MR-PRESSO global test). Furthermore, we utilized a leave-one-out approach to systematically remove individual SNPs to ascertain if any single instrumental variable was predominantly influencing the causal relationship. The findings from this method substantiated the reliability of the MR analysis, as depicted in Fig. 4B. In addition, the outcomes of the sensitivity and leave-one-out analyses for nonsignificant results are presented in Figure S2, Supplemental Digital Content, https://links.lww.com/MD/P33.

## 4. Discussion

The study conducted a MR analysis to investigate the association between circulating levels of various micronutrients and the risk of osteonecrosis of the femoral head. The study meticulously selected genetic variants as IVs based on stringent criteria to ensure robust association with the exposure (micronutrients), independence from confounders, and no pleiotropic effects on the outcome (osteonecrosis). The analysis controlled for population stratification by focusing on subjects of European ancestry and utilized a large dataset from various GWAS to enhance the generalizability and reliability of the findings. Despite the comprehensive examination of multiple micronutrients, including copper, calcium, carotene, folate, iron, magnesium, selenium, vitamins A, B12, B6, C, D, E, and zinc, no other significant associations with osteonecrosis risk were detected. The study employed multiple sensitivity analyses, such as MR-Egger regression, MR-PRESSO, and leave-one-out analysis, to ensure the robustness of the findings and to rule out potential heterogeneity or pleiotropy. The research yielded a significant positive finding regarding the role of potassium. The inverse variance weighted (IVW) method revealed that genetically predicted higher levels of potassium were associated with a reduced risk of osteonecrosis, with an odds ratio of 0.440 and a 95% confidence interval (CI) from 0.2012 to 0.959, indicating a statistically significant association at the *P*-value of .039 per 1 standard deviation. This suggests that potassium may play a protective role in the development of osteonecrosis. Emerging research has also implicated potassium as a significant contributor to skeletal integrity, although its specific relationship with osteonecrosis remains understudied.^[14]^ As an essential mineral, potassium is integral to various physiological processes, including bone metabolism.^[15]^ It is posited to influence bone density and structure by modulating the function of osteoblasts and osteoclasts, cells pivotal to bone formation and resorption.^[15]^ Additionally, potassium role in maintaining vascular health suggests a potential indirect influence on osteonecrosis by supporting bone perfusion, which is critical in preventing avascular conditions such as osteonecrosis.^[33–35]^ However, the scientific literature lacks a direct link between potassium intake and osteonecrosis risk or progression. A study among older Korean adults has indicated that higher potassium consumption correlates with greater bone mineral density and a reduced osteoporosis risk, underscoring the mineral’s beneficial impact on bone health.^[14]^ In short, while the evidence supports a positive role of potassium in bone health, its connection to osteonecrosis is not yet clearly defined, warranting further investigation.

Micronutrients including copper, calcium, carotene, folate, iron, magnesium, selenium, zinc, and vitamins A, B6, B12, C, D, and E are fundamental to bone health, but their role in osteonecrosis is not clearly defined.^[36–38]^ Copper is an essential trace element that plays a role in various biological processes, including bone metabolism. Elevated serum copper levels have been linked to increased bone mineral density and a reduced risk of osteoporosis in adults.^[39]^ Copper role in bone metabolism is suggested to be through its impact on the activity of bone-regulating cells, which could potentially influence osteonecrosis, particularly in cases of steroid-induced necrosis of the femoral head.^[36]^ However, the exact mechanisms are not yet clear and require further research. Calcium is integral to bone health, with its intake being crucial for maintaining bone mineral density and preventing bone loss and osteoporotic fractures later in life.^[40]^ Despite its well-known importance, the direct link between calcium intake and osteonecrosis risk has not been firmly established,^[33,40]^ indicating a need for more targeted research in this area. Carotene, an antioxidant abundant in fruits and vegetables, has shown promise in improving bone mineral density and reducing the risk of osteoporosis and fractures.^[41]^ The antioxidant properties of carotene may support bone health by combating oxidative stress, which is known to negatively impact bone cells. However, whether carotene can play a role in the prevention or treatment of osteonecrosis is still speculative and requires more definitive research. Folate intake has been associated with improved bone health, with cross-sectional studies showing a direct link between increased dietary folate and reduced osteoporosis risk.^[42]^ Particularly among individuals over 80 years old, a significant positive association between dietary folate intake and BMD has been observed.^[43]^ The impact of folic acid supplementation on bone metabolism, as demonstrated in a clinical trial involving postmenopausal osteoporotic women, further suggests a potential role in bone health,^[44]^ but its specific influence on osteonecrosis remains to be clarified. Iron is a critical mineral for bone health and homeostasis.^[45–48]^ Iron deficiency, which can result in anemia, may have indirect effects on bone health by affecting red blood cell production and oxygen delivery to bone tissues, potentially leading to bone loss.^[45]^ Conversely, iron overload in conditions such as hemochromatosis has been associated with bone loss, indicating a complex and delicate balance required for optimal bone health.^[47–49]^ The mechanisms by which iron levels might influence osteonecrosis are not yet fully understood and warrant further investigation. Magnesium significantly influences bone health, and its supplementation has shown potential in protecting against osteonecrosis by affecting the activity of osteoblasts and osteoclasts.^[9,50]^ Magnesium role in maintaining vascular health and its antioxidant properties may indirectly support bone perfusion and prevent conditions like osteonecrosis.^[9]^ Selenium, an essential micronutrient with potent antioxidant capabilities, has been found to exert protective effects in the context of osteonecrosis. Studies have suggested that selenium can mitigate medication-related osteonecrosis of the jaw in animal models, potentially through its antioxidant actions.^[18]^ Additionally, selenium nanoparticles integrated into hydrogels have demonstrated therapeutic benefits in treating steroid-induced osteonecrosis of the femoral head by reducing oxidative stress.^[3]^ These findings indicate that selenium may play a significant role in the management of osteonecrosis, particularly in scenarios involving oxidative damage and the use of corticosteroids. Although selenium deficiency can impact bone metabolism and increase the risk of bone disease, its specific association with osteonecrosis has not been extensively studied.^[51,52]^ Zinc is integral to bone metabolism, matrix mineralization, and osteogenesis.^[16,17]^ Zinc deficiency has been linked to impaired bone growth and development, suggesting a potential influence on the risk or progression of osteonecrosis.^[16,17]^ The indirect effects of zinc on osteonecrosis through its involvement in bone formation and matrix mineralization require further research.

Vitamins, with their diverse roles in the body, also show potential connections to bone health. Vitamin A, essential for vision, growth, and immune function, promotes early osteoblastic differentiation, which is crucial for bone formation, but excessive intake can lead to adverse effects on bone, including increased bone resorption and a higher risk of fractures.^[53,54]^ The direct link between vitamin A and osteonecrosis is not well-established, but its impact on bone metabolism could theoretically influence conditions like osteonecrosis. Vitamin B6, or pyridoxine, is an essential nutrient that affects bone metabolism, with a significant negative correlation found between serum vitamin B6 concentration and bone turnover markers in postmenopausal women with osteoporosis.^[55]^ This suggests that vitamin B6 may influence bone metabolism and could potentially affect the risk or progression of osteonecrosis,^[55]^ although the overall role of vitamin B6 in osteonecrosis is not fully understood and requires further research.^[56]^ Vitamin B12, or cobalamin, is involved in DNA synthesis, red blood cell formation, and nervous system maintenance.^[57]^ Its indirect role in bone metabolism is suggested through its involvement in homocysteine metabolism.^[57,58]^ Elevated homocysteine levels, associated with vitamin B12 deficiency, can lead to decreased bone mineral density and an increased risk of fractures,^[57,58]^ indicating the importance of adequate vitamin B12 levels for bone health. Vitamin C, with its antioxidant properties, is recognized for its importance in collagen synthesis, a key component of bone matrix, and may influence osteoblast and osteoclast activity, thus affecting bone remodeling.^[9,59,60]^ Its potential role in attenuating steroid-associated osteonecrosis and promoting bone repair requires further investigation. Vitamin D is crucial for bone health, and its deficiency has been linked to osteoporosis and osteomalacia.^[8,61]^ While the direct relationship between vitamin D and osteonecrosis is not extensively explored, imbalances in vitamin D and calcium levels, which can occur with bisphosphonate treatment, may be associated with osteonecrosis of the jaw.^[62]^ Vitamin E, with its antioxidant and anti-inflammatory properties, has been suggested to protect against bone loss and may play a role in the prevention and treatment of osteonecrosis.^[4,63]^ Its potential protective effects on the femoral head in rats, reducing oxidative stress, inflammation, and microvascular dysfunction, indicate promise but necessitate further research.^[4,64]^ In short, the relationship between micronutrients and osteonecrosis is multifaceted and not fully understood. The potential protective effects of certain micronutrients, as well as their roles in bone metabolism, oxidative stress, and inflammation, suggest important avenues for future research. A more comprehensive understanding of these relationships could provide valuable insights into the prevention and treatment of osteonecrosis, emphasizing the need for continued investigation.

Given the observed potential protective role of potassium in ONFH, further research is needed to elucidate the underlying mechanisms. In vitro and in vivo studies could be conducted to investigate how potassium affects osteoblast and osteoclast function, bone metabolism, and vascular health. Understanding the molecular pathways involved could provide potential targets for therapeutic interventions. For other micronutrients that have been implicated in bone health but showed inconclusive results in our study, mechanistic studies are also warranted. This could involve exploring their effects on bone cell signaling, gene expression, and oxidative stress, as well as their interactions with other factors involved in ONFH pathogenesis.

The research, while insightful, encounters several inherent limitations. Principally, the European derivation of the dataset from which the study was drawn may impede the broad applicability of the findings to diverse populations. Additionally, the scope of the study is potentially constrained by the finite number of case and control samples with osteonecrosis. Considering the subtle influence of genetic variance on exposure, which demands extensive cohorts for the attainment of statistically robust outcomes, the modest size of our dataset could be a confining factor. Moreover, the study’s credibility is not fortified by cross-validation against additional databases, which could affect the generalizability of our results. Finally, the publicly accessible aggregated meta-analyses were deficient in thorough demographic details, precluding the execution of stratified analyses predicated on sex and age. This shortfall also obstructed a holistic evaluation of the potential nonlinear dynamics between micronutrient levels and osteonecrosis, suggesting that the study’s conclusions may not encapsulate the full spectrum of this complex relationship.

## 5. Conclusions

While the study did not find a causal link between most of the examined micronutrients and osteonecrosis, the significant association of potassium with a reduced risk of the condition highlights the importance of considering micronutrient levels in the prevention and management strategies for osteonecrosis. Further research is needed to elucidate the underlying mechanisms and to explore the potential therapeutic applications of modulating potassium levels in patients at risk of osteonecrosis.

## Author contributions

**Conceptualization:** Rui Huang, Xue Li.

**Data curation:** Hai Hu, Pengfei Liu, Shujun Ren.

**Funding acquisition:** Hai Hu.

**Methodology:** Hai Hu, Pengfei Liu.

**Software:** Rui Huang, Xue Li, Pengfei Liu.

**Supervision:** Shujun Ren, Yiwei Shen.

**Writing – original draft:** Hai Hu, Rui Huang, Xue Li, Pengfei Liu, Shujun Ren, Yiwei Shen.

**Writing – review & editing:** Rui Huang, Xue Li, Shujun Ren, Yiwei Shen.

## Supplementary Material


