# Supplementary material for: Genetic determinants of micronutrient levels and their causal impact on osteonecrosis of the femoral head: A 2-sample Mendelian randomization study
Source: Medicine (Baltimore). 2025 May 30;104(22):e42487. doi: 10.1097/MD.0000000000042487 (PMC12425091; doi:10.1097/MD.0000000000042487)

Supplementary figure 1 Causal correlations of copper, calcium, carotene, folate, iron, magnesium, selenium, vitamin A, vitamin B12, vitamin B6, vitamin C, vitamin D, vitamin E, and zinc on osteonecrosis. The figure shows no significant associations between these micronutrients and osteonecrosis risk as determined by the relevant analyses in the study.

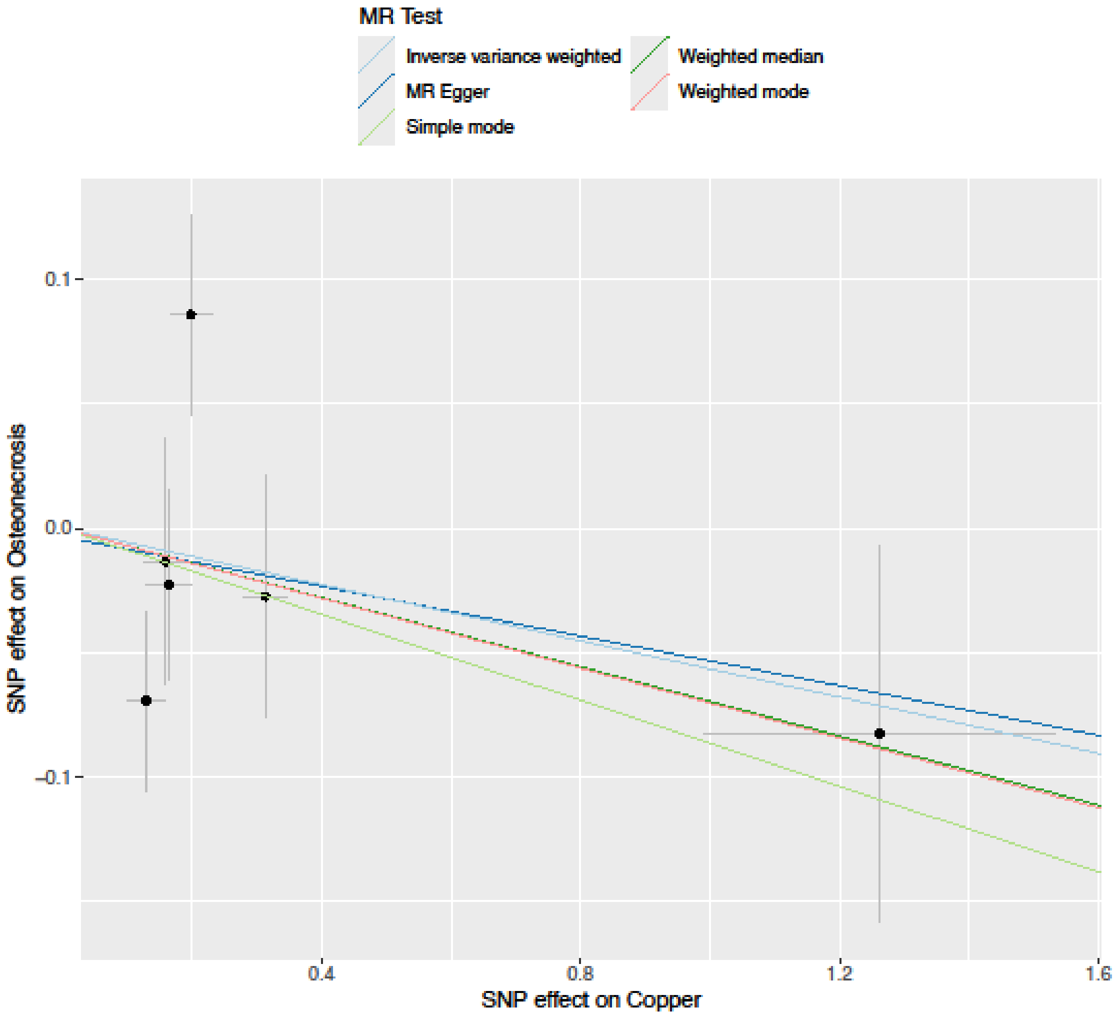

Supplementary figure 2 Leave - one - out analyses for non-significant results of micronutrients other than potassium on osteonecrosis.

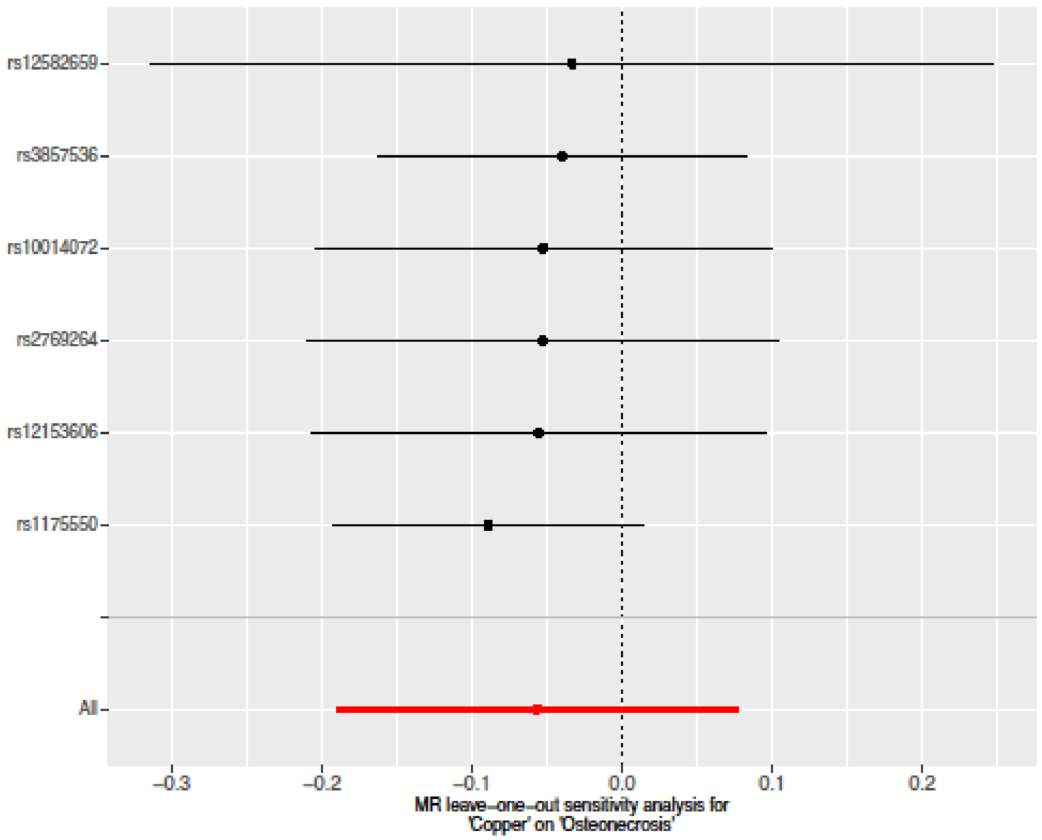

Supplement: Supplementary file 2 [file medi-104-e42487-s002.pdf]
